# Natural Biological Properties Inherited from Native Endemic Flora in Honeys from Lake Ranco Area of Southern Chile: A Botanical and Physicochemical Approach

**DOI:** 10.3390/molecules30193984

**Published:** 2025-10-04

**Authors:** Enrique Mejías, Carlos Gómez, Pablo Díaz, Tatiana Garrido

**Affiliations:** 1 Centro de Tecnologías Nucleares en Ecosistemas Vulnerables, División de Investigación y Aplicaciones Nucleares—Comisión Chilena de Energía Nuclear, Nueva Bilbao 12501, Las Condes, Santiago 7600713, Chile; 2 Departamento de Química Inorgánica y Analítica, Facultad de Ciencias Químicas y Farmacéuticas, Universidad de Chile, Olivos 1007, Independencia, Santiago 8391063, Chile; 3 Facultad de Medicina y Ciencia—Campus Los Leones, Universidad San Sebastián, Carmen Silva 2444, Providencia, Santiago 7510156, Chile

**Keywords:** Chilean honey, botanical origin, antioxidants, phenolic compounds, HMF

## Abstract

Chile boasts a rich variety of native endemic melliferous flora, recognized internationally for the excellent taste and biological properties of its honeys. While honey production occurs across various regions, the southern zone, particularly near native rainforests, yields highly valued honeys that often lack comprehensive analytical characterization. This study was focused on seven apiaries near Lake Ranco in the Los Rios Region, collecting two honey samples from each location over two consecutive harvesting seasons, totaling 20 samples. Key parameters analyzed included botanical origin, total carbohydrates, glucose/fructose ratio, total phenolic compounds, antioxidant/antiradical activity and 5-hydroxymethylfurfural (HMF) content. The results indicated a significant influence of three native species, *Eucryphia cordifolia*, *Caldcluvia paniculata*, and *Weinmannia trichosperma*, on the antioxidant activity of the honeys. The physicochemical parameters measured, along with the concentration and activity of the compounds responsible for this activity, establish a very characteristic pattern for the monofloral honeys of these three species. This information could serve as a foundation for constructing a map to help differentiate Chilean honeys based on their natural biological attributes helpful for consumer health, generating distinctive profiles that would contribute to accurately guaranteeing their geographical origin and, consequently, increase their specific value.

## 1. Introduction

Globally, various regulations and standards have been established to ensure the preservation and care of bees, acknowledging their crucial role in forest conservation and pollination, which is vital to produce plant-based food [1]. Additionally, honey production and the regulations pertaining to proper honey labeling are ongoing topics of discussion [2]. These regulations promote the sustainability of beekeeping, safeguarding not only the bees but also the producers and consumers of hive products, such as honey and others [3,4]. By enforcing these protocols, we can maintain the balance of ecosystems while supporting the apiculture industry [5].

Human activities and climate change pose a persistent threat to bees, placing beekeeping in an uncertain scenario [6]. Bees are impacted in various ways depending on the specific region or geographical area, making it challenging to develop a universal set of actions applicable to all beekeepers globally [7]. This variability in challenges necessitates tailored approaches that address local conditions to ensure the health of bee populations and the sustainability of beekeeping practices [8,9].

In this context, recent years have seen a connection between the resilience of beehives and the presence of apiaries situated in areas with minimal or no human intervention [10]. While honeybees are recognized as the most efficient pollinators, their role in agriculture also poses significant risks due to exposure to pesticides and agrochemical compounds [11]. This duality highlights the importance of creating safe environments for bees, avoiding exposure to pesticides and agrochemical compounds [12].

In this context, the presence of melliferous plants and beehives situated in pollutant-free areas is essential for producing honey and other bee products that adhere to safety standards, ensuring their safe consumption and maintaining their natural properties [13,14]. In accordance with its composition, honey is regarded as a functional food, because it includes nutrients, trace elements, and vitamins [15,16]. Notably, it contains phenolic compounds and flavonoids derived from the secondary metabolism of the melliferous species from which bees collect nectar [17]. These compounds impart various biological properties to honey, promoting the health of individuals who consume it regularly [18]. Thus, these compounds are primarily linked to the botanical origin of the honey [19] and correspond to a diverse family of molecules that exhibit variable chromatographic profiles [20,21]. Additionally, they are associated with the color intensity [22,23] and flavor of honey [24,25].

These attributes vary based on the geographical area of production [26] and the presence of other organic components, such as methylglyoxal in manuka honey [27], or the mineral element profile [28]. Together, these factors classify honey according to its potential medical applications resulting from ingestion or use [29]. Regular and continuous intake over time can lead to various effects that modify metabolic parameters in individuals, including a reduction in body mass index in overweight individuals and improvements in lipid profiles [30]. Additionally, honey offers benefits in wound treatment [31], anti-inflammatory effects [32], antibiotic activity [33], antioxidant properties [34], and complementary uses in cancer treatment [35].

Chile has a diverse native forest across its territory, hosting numerous melliferous species that serve as vital food sources for bees [36]. The pollination activities of these bees play a crucial role in maintaining forest biodiversity through their ongoing interactions with flora. This symbiotic relationship not only supports the ecosystems but also enhances honey production [37]. In this respect, Chilean honeys from endemic native species possess interesting biological properties inherited from the specific floral source of origin [38].

The Chilean regulation (law) No. 21,489 on the Promotion, Protection, and Encouragement of Beekeeping, enacted in 2022, acknowledges the significance of the national beekeeping sector by the State, particularly for its contribution to the diversity and quality of a substantial portion of agricultural production systems [39]. This law establishes the promotion, protection, and encouragement of the sustainable development of beekeeping as a forestry and farming activity by regulating the production and extraction of bee products, the commercialization of bee biological material, and pollination services from all beehives within the national territory.

Likewise, according to data published by the Office of Agricultural Studies and Policies (ODEPA) in its interactive beekeeping bulletin report, Chile exports a significant portion of its honey production. In 2024, the same government agency reported that Chile exported over seven thousand tons of honey, while so far in 2025, the amount of honey exported has already exceeded three thousand five hundred tons [40].

Thus, 20 honeys obtained from apiaries located in the area surrounding Lake Ranco in the town of Futrono in southern Chile (Los Ríos Region) were analyzed for this study. From a botanical point of view, this region is characterized by a high endemism of native melliferous species that give rise to honeys recognized for their distinctive flavour, colour and texture. The proposed objective was to characterize physicochemical parameters, antioxidant activity and quality descriptors to correlate the data according to the botanical origin determined by melissopalynology.

## 2. Results

### 2.1. Botanical Origin of Honey Samples

The botanical and geographical origins of the honey samples used in this study are summarized in Table 1. It is important to emphasize that the analysis exclusively concentrated on monofloral honeys derived from native endemic species, adhering to the classifications established by current Chilean regulations [41]. This set of samples was selected from a larger group of honeys produced in the same geographical area. After analysis, only monofloral honeys with a high percentage of monoflorality were chosen to minimize dispersion and enhance the interpretation of subsequent results.The data includes both the scientific names of the predominant native species and their corresponding common names, thereby ensuring clarity and enhancing the understanding of each honey type from the area surrounding Lake Ranco in Futrono. After analyzing the honey samples, Tiaca (*C. paniculata*), Ulmo (*E. cordifolia*), and Tineo (*W. trichosperma*) were identified. These meliferous species are commonly found in the native forests of the southern Chilean landscape.

### 2.2. Carbohydrate Content in Honey Samples

**Figure 1:**
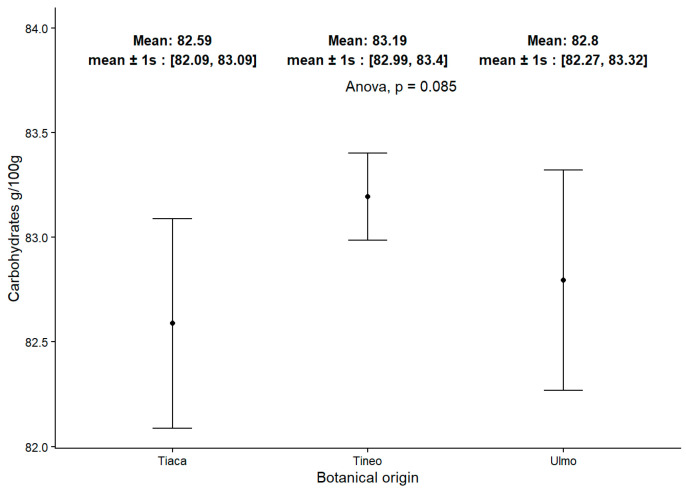
Total Carbohydrate content in samples considering the botanical origin of honeys.

Total carbohydrate content was assessed in the set of honeys to further correlate the obtained values with the botanical origin of these samples, as shown in Figure 1. The results are presented by considering three groups of honeys defined after determining the pollen residue composition of the samples and their respective total carbohydrate presence. The determination of carbohydrates for honeys produced in this area showed a similar range to that described for honeys from similar species [42]. In this regard, the amount of total carbohydrates and moisture content remained consistent in honeys from the area near Lake Ranco, regardless of botanical composition, indicating a stable nutrient profile characteristic of honeys from this geographical region. The results obtained here reaffirm this trend.

### 2.3. Glucose and Fructose Content in Honey Samples

**Figure 2:**
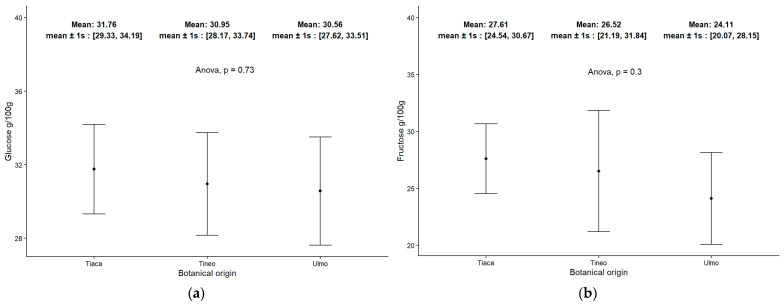
Monosaccharides pattern in monofloral honey samples from Lake Ranco zone: (**a**) Glucose and (**b**) Fructose.

The identification of the two most detected sugars in honey is illustrated in the graphs of Figure 2a for glucose and Figure 2b for fructose, corresponding to the samples analyzed in this study. In both cases, the results are related to the three groups of honeys based on their botanical origin. Beyond the liquid/solid state of the honeys, the data obtained complement the total carbohydrate content mentioned in the previous section and are consistent with the recorded values for total carbohydrates. Moreover, the pattern of sugars in all groups of monofloral honeys indicated a slightly higher content of glucose compared to fructose. In Tineo (*W. trichosperma*) honey samples, both sugars appeared in nearly similar ratios, whereas in Tiaca (*C. paniculata*) and Ulmo (*E. cordifolia*) honeys, this difference was more pronounced.

### 2.4. Content of Total Phenolic Compounds in Monofloral Honey Samples

**Figure 3:**
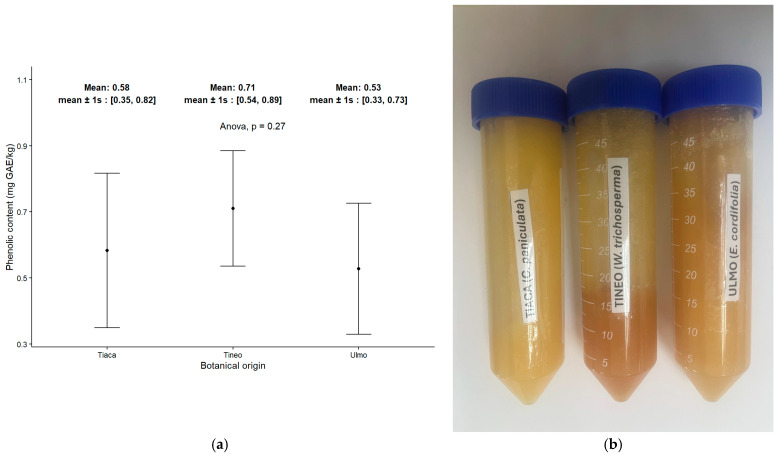
Bioactive compounds in honey samples. (**a**) Total Phenolic compound content. (**b**) Color intensity of honey samples in relation to botanical origin.

The content of total phenolic compounds in the set of honeys is illustrated in Figure 3. Regardless of botanical origin, similar trends were observed in the amounts of these compounds across the groups. Although the percentage of botanical predominance within each group varies, as shown in Table 1, this variation may lead to a significant degree of dispersion in the subsequent data from further analyses. However, the subtle changes in color intensity from one group of samples to another suggest that the differences are primarily related to the chemical nature of these compounds, despite the similar content found in honeys with different botanical compositions.

### 2.5. Antiradical and Antioxidant Activities of Monofloral Honeys

**Figure 4:**
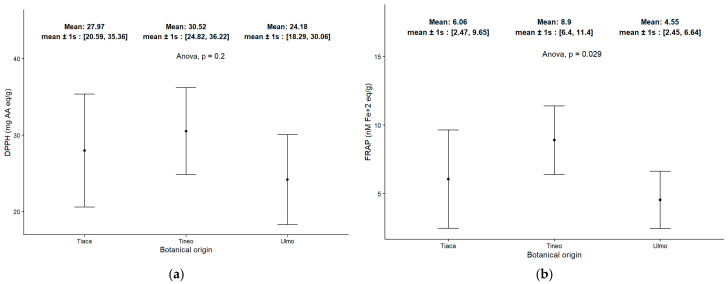
Antioxidant power assessed in honey samples by two complementary methods: DPPH assay (**a**) and FRAP assay (**b**).

An analysis of the antioxidant capability of honey samples is presented using two complementary parameters, as seen in the DPPH assay (Figure 4a) and the FRAP values (Figure 4b). The results are consistent and confirm the presence of antioxidant strength assessed by different chemical mechanisms. Through these complementary methods, Tineo (*W. trichosperma*) honey samples ranked highest on average in terms of antioxidant power, while Ulmo (*E. cordifolia*) honey demonstrated decreased activity compared to the other two groups. Ulmo is known for its antibacterial capability; thus, this decreased antioxidant attribute is expected according to prior studies [43,44].

### 2.6. 5-Hydroxymethylfurfural (HMF) Content in Honey

**Figure 5:**
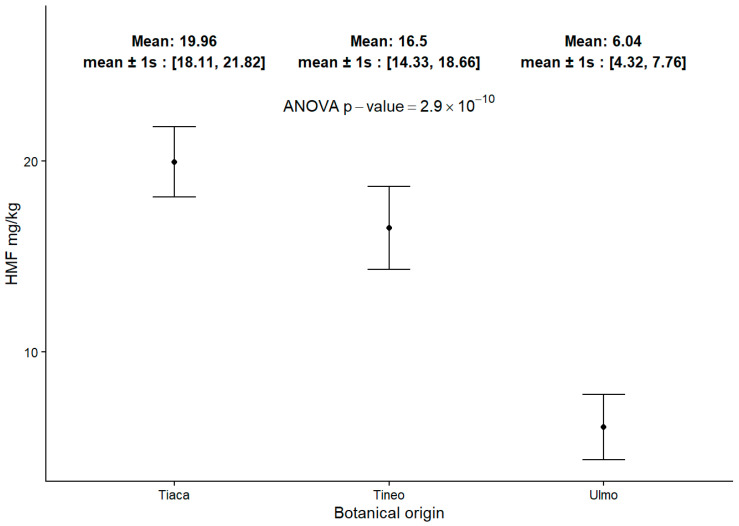
HMF content in monofloral honey samples.

The analysis of the HMF content is shown in Figure 5. In this case, the differences among the groups are well defined and demonstrate a decreasing trend, starting with higher content in Tiaca (*C. paniculata*) honeys, a moderate presence in Tineo samples, and the last group with the lowest content corresponding to Ulmo (*E. cordifolia*) honeys. Despite the observed differences among all three groups of honey, all samples have acceptable levels of this compound, according to international regulations and recommendations [45].

## 3. Discussion

One of the advantages of native forests is their ability to maintain a stable microclimate, ensuring a consistent water balance. This stability helps preserve slopes and soils, protects habitats for wildlife and plants, and supports the development and maintenance of the landscape [46]. Given Chile’s varied geography, native forests with a diverse array of plant species can be found throughout the country. Moreover, these native forests are home to numerous melliferous species that provide food for both bees and native bees. Additionally, their pollinating actions contribute to maintaining forest biodiversity through constant interactions, which also enhance honey production [47,48]. In that way, at least four forest formations are generally recognized, with the evergreen forest [36,49] being particularly prominent in the southern regions. This formation describes the characteristics of the Valdivian Forest, which is rich in species such as Tiaca (*C. paniculata*), Tineo (*W. trichosperma*), and Ulmo (*E. cordifolia*), key contributors to the botanical origins of the honeys analyzed in this study.

It is important to highlight that beekeeping is practiced throughout Chile, although the productive area begins in the Atacama Region and extends to the Aysén Region. According to the latest records published by the Agricultural and Livestock Service, SAG [50], up to 2024, there were 10,504 beekeepers in Chile managing 1,404,214 hives, with about 25% concentrated in the southern part of the country, where the town of Futrono is located, the origin of the honeys studied here. This area is notable for its high endemism of honey-producing species, resulting in honeys with unique flavors, textures, and biological properties. In this context, Chilean honeys derived from endemic native species possess intriguing biological properties inherited from their specific floral sources [51].

In this study, we characterized monofloral honeys from native species that originate from the same geographical area and are produced throughout the season according to the specific flowering calendars of each species. All three species belong to the Cunoniaceae family and exhibit morphological similarities, as they are evergreen trees with dense, thick, and bright green foliage. Various studies have concluded that Ulmo honey (*E. cordifolia*) exhibits antibiotic activity, while Tiaca honey (*C. paniculata*) demonstrates biological activity related to antioxidant capacity. However, despite the available information, this study offers insights into Chilean honeys of commercial interest produced in the same locality. Notably, only some of the data obtained for the physicochemical parameters allowed for establishing differences associated with their botanical origin and, consequently, their geographical origin. Other components of the total nutritional content were found to be conserved among the different samples, with no significant differences observed. In the group of honeys analyzed, no significant differences were found in % ash, % moisture, or sodium content, indicating a stable composition for honeys produced in this area.

**Figure 6:**
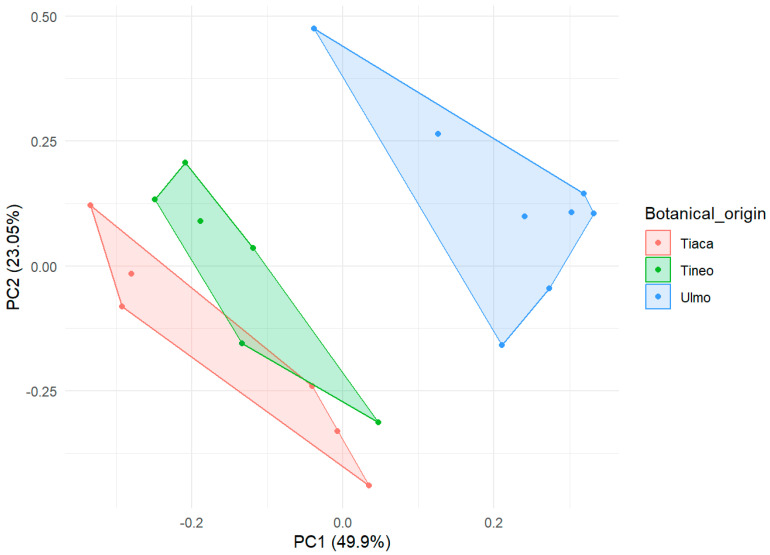
Principal component analysis (PCA) for monofloral honey samples.

However, as shown in Figure 6, Principal Component Analysis (PCA) reveals trends that create specific clusters for the three types of honey studied here. These differences are primarily attributed to the presence of phenolic compounds responsible for biological activity and variations in glucose/fructose ratios detected in the analyses. These differences correspond with the distinct organoleptic properties of each type of honey and help define their specific color tones. Additionally, this study is novel in its characterization of Tineo honey (*W. trichosperma*), a common species in southern Chile that produces abundant amounts of honey but has been little studied regarding its composition and potential biological attributes.

On the other hand, HMF is a compound that occurs naturally in low concentrations in honey as a product of the reaction between its components, such as sugars, phenolic compounds, and organic acids, and serves as an indicator of honey freshness [52]. Similarly, the synthesis of this compound in honey is accelerated when it is improperly heated, which can happen when honey is manipulated to alter its organoleptic aspects after harvesting. The formation of this compound tends to increase in honeys with a higher proportion of keto hexoses, such as fructose [53].

Likewise, in organisms, HMF is degraded through a series of reactions that involve the oxidation of an aldehyde group and subsequent binding to a glycine molecule, allowing the metabolites derived from this compound to be eliminated in urine. Although there is no in vitro evidence of genotoxicity, intestinal lesions have been observed in rats due to their presence [54].

In this regard, the regulation establishes limits for HMF, stating that this compound must not exceed 40 mg/kg of honey due to its potential carcinogenic effects [45]. However, data suggest that, in low quantities, HMF may serve an opposing role by acting as an antioxidant [55]. Given the low concentrations in which HMF is typically found in honey, its presence may complement the antioxidant action of the phenolic compounds present in any given honey.

Thus, the results of this study allow for the differentiation of the three groups of honeys based on the quality descriptor parameters measured. The evidence suggests that, beyond the quantity of phenolic compounds in each group’s composition, the differences lie in the type of compounds and their associated properties. This explains the variations in biological activities observed in Chilean honeys from both the south and other productive regions of the country [56]. In this context, the differences in HMF detection among these honeys could serve as an indirect indicator of the profiles that these compounds possess for each honey, facilitating their differentiation and specific characterization. This aligns with the results from Pearson correlations between HMF and total phenolic compounds, where Tiaca (*C. paniculata*) honey showed R = 0.75; *p* = 0.088, while Ulmo (*E. cordifolia*) honey indicated R = 0.58; *p* = 0.087. The same statistical analysis found no correlations between HMF content, and the amount and type of sugars detected in each honey group. Therefore, the results of this study indicate that, although it is not possible to establish a protocol for detecting fraudulent honeys using physicochemical profiles, it is feasible to define a pattern that, in conjunction with other techniques, can create a comprehensive identification tool for Chilean honeys that incorporates these data.

For decades, plants and forest products have been studied for their beneficial substances for human and animal health. Traditional knowledge has linked certain characteristics of honey to the treatment of respiratory and digestive issues, as well as its relaxing and invigorating effects due to its nutritional composition [57]. Additionally, various studies on food matrices highlight honey as an excellent source of natural antioxidants, mainly due to the phenols and flavonoids derived from the specific floral sources that produce honey and bee products. These compounds help protect tissues against free radicals, slowing down aging and the premature deterioration of organs and tissues [58]. Another interesting attribute of honey is its antibacterial activity, which is associated with its high osmolarity, elevated acidity (low pH), and the presence of hydrogen peroxide in its composition [59].

These natural attributes change from one geographical area to another, resulting in a wide variety of honeys that still require thorough characterization, such as the set of samples examined in this study. While Chilean honey is well-regarded in the international markets where it is exported, there remains a lack of detailed differentiation despite the diversity of Chilean native endemic monofloral and polyfloral honey produced and their potential benefits. The findings of this study enable us to identify at least three native honeys endemic to Chile that exhibit promising antioxidant properties correlated with their floral origins, and physicochemical parameter (*W. trichosperma* has not been studied in detail until now) along with distinct colors and textures to attract any honey consumer.

The biological characterization linked to organoleptic attributes will facilitate the establishment of profiles based on their botanical and geographical origins. This approach will increase the prestige that Chilean honey has reached thus far and increase its added value by offering additional health benefits with scientific evidence to regular consumers both in Chile and around the world.

## 4. Materials and Methods

### 4.1. Honey Samples

Twenty honey samples were collected from five apiaries located near Lake Ranco, Futrono, in the Los Ríos Region of Chile (−40.201386, −72.266359), during two consecutive harvest seasons corresponding to the summers of 2023 and 2024. All the apiaries were surrounded by native forests and situated far from agricultural production areas. The samples were contained in amber glass bottles and transported to the laboratory in a portable cooler. Upon arrival, the samples were stored in a dark room at 10 °C until analysis were performed.

### 4.2. Mellisopalynological Analysis

The botanical composition of honey samples was quantitatively analyzed using methods described by Louveaux [60]. In brief, 20 g of honey was placed on acetolyzed slides [41]. A sample aliquot was then diluted with 20 mL of warm distilled water at 40 °C and transferred to a suitable tube, which was centrifuged at 3500 rpm for 10 min. The supernatant was discarded, leaving the pollen residue at the bottom of the tube, which was subsequently resuspended in 100 µL of distilled water. An aliquot of 20 µL was taken and mixed with 10 µL of Calberla’s solution, using either basic fuchsine or diamond stain. The slide was gently dried, and then 15 µL of melted glycerinated gelatin was added to the mixture. For each sample, pollen grain residues were identified using an optical microscope at magnifications of 400× and 1000×.

### 4.3. Total Carbohydrate Content in Honey Samples

The total carbohydrate percentage in each honey sample was initially determined using refractometry, expressed as a weight/weight (*w*/*w*) percentage. For further analysis, 15 g of honey was precisely weighed and then mixed with 10 mL of water to create a solution. The pH of this solution was adjusted to 1.0 by the addition of 1.2 M hydrochloric acid (HCl), utilizing an automatic titrator equipped with a combined pH electrode for accuracy [61]. Later, the total carbohydrate concentration was decreased to 40.0% (*w*/*w*) by dilution with acidified water (pH = 1.5). It is noteworthy that the average total carbohydrate content in pure honey is typically around 80% *w*/*w*, indicating that the dilution process was essential for the appropriate handling of the samples in the analytical procedures [62]. This controlled adjustment allows for reliable comparisons and assessments of carbohydrate profiles in the honey samples analyzed.

### 4.4. Glucose/Fructose Determination by LC-MS/MS

The analysis of honey samples using UPLC–MS/MS was conducted with a XEVO Triple Quadrupole Tandem Mass Spectrometer (Milford, MA, USA), integrating methods based on previously detailed protocols with adjustments [63]. The separation process utilized an Acquity Ethylene Bridged Hybrid (BEH) C18 column (Waters Corp, Milford, MA, USA), characterized by its dimensions (130 Å, 1.7 µm, 2.1 mm × 50 mm). The mobile phase was a gradient of 95% acetonitrile (ACN) and 0.05% formic acid (FA) alongside 90% HPLC grade water, ensuring all solutions were formulated with HPLC grade water for consistency.

The analysis was performed at an oven temperature of 37 °C, with a sample injection volume of 5 µL. The MS/MS parameters set for the analysis included a positive ionization mode, MRM scan type, a dwell time of 15 ms, an ion spray voltage of 5500 V, a source temperature of 300 °C, and a total analysis duration of 20 min [64].

### 4.5. Honey Bioactive Compounds Extraction for Colorimetric Assays

Five grams of honey were dissolved in 10.0 mL of distilled water to create a homogeneous solution after vortexing.

The extraction of bioactive compounds from honey was conducted according to the method outlined by Paula et al. and [65,66], with several modifications. A manifold chamber (WATERS) and Solid Phase Extraction (SPE) columns (C18 Oasis HLB 30 µm–6 cc/500 mg) were employed for this extraction process.

To activate the SPE-C18 columns, 10 mL of methanol was initially applied, followed by 10 mL of dichloromethane, and finally, 10 mL of deionized water. This sequence of solvents ensures that the columns are properly conditioned for effective analyte retention. After activation, the entire volume of each honey solution was passed through the SPE column. The column was then washed with 10 mL of dichloromethane, followed by 10 mL of deionized water. Purified phenolic compounds were subsequently extracted using 4 mL of methanol. The final bioactive compound extracts (BCE) solutions were stored in dark vials at room temperature until the colorimetric analyses were performed. The pH of the solutions ranged from 5.0 to 6.5 [42].

### 4.6. Total Phenolic Compounds Content

The determination of phenolic content in honey samples was carried out following the method established by Singleton and Rossi [67], with adaptations specific to honey [68]. Two hundred microliters (200 µL) of each BCE solution were measured and mixed with 50 mL of Folin–Ciocalteu reagent (Merck, Darmstadt, Germany) to initiate the reaction. Following this, 150 µL of 20% sodium carbonate (Na_2_CO_3_) solution (Merck) was added to the mixture, which promotes the development of color indicative of phenolic compounds. Distilled water was then added to bring the total volume of the solution to 1.00 mL, ensuring proper dilution for measurement. The mixture was incubated for 30 min to allow for the reaction to occur, facilitating the formation of a blue complex that can be measured spectrophotometrically. After incubation, the absorbance of the solution was measured at a wavelength of 765 nm using a spectrophotometer.

A calibration curve was generated using gallic acid (Sigma-Aldrich, St. Louis, MO, USA) as the standard, with concentrations ranging from 0 to 150 mg/mL The results obtained from the analysis were expressed as Total Phenolic content, reported in milligrams of gallic acid equivalent per kilogram of honey sample (mg GAE/kg).

### 4.7. DPPH Assay for Antiradical Activity of Honey Samples

The antiradical activity of honey was evaluated using the DPPH (1,1-diphenyl-2-picrylhydrazyl) assay, following a combination of the procedures by Meda et al. [69] and Dimitriu et al. [70]. For each sample, a total of 250 µL of the BCE solution was mixed with 750 µL of a DPPH solution in methanol (0.02 mg DPPH/mL). The mixture was allowed to react for 15 min, after which the absorbance was measured at 517 nm using a spectrophotometer. A blank sample was prepared with methanol to account for background absorbance. Ascorbic acid (Calbio-Chem, Darmstadt, Germany) served as a standard to generate a calibration curve (1–10 mg/mL). The results for antiradical activity were expressed as milligrams of ascorbic acid equivalents per gram of sample (mg AA eq/g).

### 4.8. FRAP Assay for Antioxidant Activity

FRAP assays were conducted following the protocols established by Bertoncelj et al. [71] and the adapted modifications by Mejías et al. [42]. In summary, the FRAP reagent was prepared by combining 2.5 mL of 2,4,6-tripyridyls-triazine (TPTZ; 10 mM TPTZ in 40 mM HCl) with 2.5 mL of 20 mM FeCl_3_. Subsequently, 25.0 mL of 0.3 M acetate buffer (pH 3.6) was added to the mixture. The FRAP reagent was freshly prepared for each assay to ensure optimal results. To assess the antioxidant capacity, 0.200 mL of the BCE solution was mixed with 1.8 mL of the FRAP reagent. After a 10-min incubation, the absorbance was measured at 593 nm. Iron sulfate heptahydrate (FeSO_4_·7H_2_O) was utilized as the standard for constructing a calibration curve, with concentrations ranging from 50 to 1000 µM. The results were expressed as millimoles of Fe^2+^ equivalents per gram of sample (mM Fe^2+^ eq/g).

### 4.9. Determination of 5-Hydroxymethylfurfural (HMF) in Honey

To determine the HMF content in honey samples, the method outlined by Nobari Moghaddam et al. [72] was adapted with modifications. Initially, 1.00 g of honey sample was accurately weighed in duplicate into 15.0 mL Falcon tubes. Subsequently, 5 mL of distilled water was added to each tube, and the mixture was shaken vigorously to ensure complete dissolution of the honey.

In the next step, the Carrez Clarification Kit (Merck KGaA, Darmstadt, Germany) was employed. To each tube, 100 µL of Carrez I solution was added, followed by the addition of 100 µL of Carrez II solution. The mixture was then transferred to a volumetric flask, and distilled water was added to achieve a final volume of 10.0 mL. Following this, the SPE-C18 extraction system described in Section 4.5 was employed, utilizing only deionized water to elute the extracts containing HMF.

Finally, 5 mL of each extract was transferred to new 15.0 mL Falcon tubes, resulting in two tubes per sample. To one of these tubes, 2.5 mL of sodium bisulfite was added, while 2.5 mL of water was added to the other. Absorbances at 284 nm and 336 nm were then measured, and calculations were performed considering the new volumes in accordance with the factor specified in method 980.23 AOAC (1995). The results were expressed as milligrams of HMF per kilogram of honey (mg/kg).

### 4.10. Statistical Analysis

An exploratory data analysis based on statistical graphics was conducted to select an appropriate analysis and check assumptions. One-way analysis of variance (ANOVA) was employed to compare the physicochemical characteristics based on their botanical origin (phenolic compound content, DPPH and FRAP assays, glucose, etc.) with α = 0.05. A Pearson correlation analysis was performed for all numerical variables. Additionally, a principal component analysis was executed to reduce dimensionality, and a cluster analysis was conducted to group the different samples analyzed. All data were processed using R software version 4.5.0—R Development Core Team 2025. Graphs were generated using the ggplot2 package version 4.0.0.

## 5. Conclusions

The detection of physicochemical attribute profiles enables the characterization of monofloral honeys derived from native Chilean endemic species of commercial interest, produced in regions that also exhibit notable biological characteristics, including health benefits for consumers of these apicultural products. The development of databases with analytical data that verify the authentic origin of honey serves as a powerful tool in combating food counterfeiting and honey fraud. However, it is essential to complement the information obtained with more advanced techniques that can provide deeper insights into the unique characteristics of honeys, particularly those that are distinctive to Chile.

## Figures and Tables

**Table 1:** Geographical and botanical origins of monofloral honeys are detailed in this study. The data is presented with both the scientific and common names, along with the percentage of the main species identified through mellisopalynological analysis.

		Predominant Botanical Species		
Sample	Geographical Origin	Scientific Name	Common Name	Predominance (%)
1	Futrono Futrono	*Caldcluvia paniculata*	Tiaca	63
2	Futrono Futrono	*Caldcluvia paniculata*	Tiaca	71
3	Cerrillos Futrono	*Eucryphia cordifolia*	Ulmo	88
4	Llifén Futrono	*Weinmannia trichosperma*	Tineo	58
5	Llifén Futrono	*Weinmannia trichosperma*	Tineo	61
6	Cerrillos Futrono	*Eucryphia cordifolia*	Ulmo	93
7	Santa Juana Futrono	*Caldcluvia paniculata*	Tiaca	78
8	Futrono Futrono	*Eucryphia cordifolia*	Ulmo	86
9	Cerrillos Futrono	*Eucryphia cordifolia*	Ulmo	79
10	Llifén Futrono	*Weinmannia trichosperma*	Tineo	55
11	Llifén Futrono	*Caldcluvia paniculata*	Tiaca	68
12	Futrono Futrono	*Weinmannia trichosperma*	Tineo	54
13	Santa Juana Futrono	*Eucryphia cordifolia*	Ulmo	95
14	Llifén Futrono	*Eucryphia cordifolia*	Ulmo	84
15	Futrono Futrono	*Eucryphia cordifolia*	Ulmo	89
16	Futrono Futrono	*Weinmannia trichosperma*	Tineo	52
17	Santa Juana Futrono	*Caldcluvia paniculata*	Tiaca	77
18	Cerrillos Futrono	*Caldcluvia paniculata*	Tiaca	71
19	Llifén Futrono	*Weinmannia trichosperma*	Tineo	60
20	Cerrillos Futrono	*Eucryphia cordifolia*	Ulmo	91

## Data Availability

The original contributions presented in the study are included in the article, further inquiries can be directed to the corresponding author.
